# Patient Satisfaction with Methadone Maintenance Treatment in Vietnam: A Comparison of Different Integrative-Service Delivery Models

**DOI:** 10.1371/journal.pone.0142644

**Published:** 2015-11-10

**Authors:** Bach Xuan Tran, Long Hoang Nguyen, Huong Thu Thi Phan, Carl A. Latkin

**Affiliations:** 1 Institute for Preventive Medicine and Public Health, Hanoi Medical University, Hanoi, Vietnam; 2 Johns Hopkins Bloomberg School of Public Health, Baltimore, Maryland, United States of America; 3 School of Medicine and Pharmacy, Vietnam National University, Hanoi, Vietnam; 4 Authority of HIV/AIDS Control, Ministry of Health, Hanoi, Vietnam; Medical University of Vienna, AUSTRIA

## Abstract

**Background:**

Patient satisfaction is an important component of quality in healthcare delivery. To inform the expansion of Methadone Maintenance Treatment (MMT) services in Vietnam, we examined the satisfaction of patients with regards to different services delivery models and identified its associated factors.

**Methods:**

We interviewed 1,016 MMT patients at 5 clinics in Hanoi and Nam Dinh province. The modified SATIS instrument, a 10-item scale, was used to measure three dimensions: “Services quality and convenience”, “Health workers’ capacity and responsiveness” and “Inter-professional care”.

**Results:**

The average score was high across three SATIS dimensions. However, only one third of patients completely satisfied with general health services and treatment outcomes. Older age, higher education, having any problem in self-care and anxiety/depression were negatively associated with patient’s satisfaction. Meanwhile, patients receiving MMT at clinics, where more comprehensive HIV and general health care services were available, were more likely to report a complete satisfaction.

**Conclusion:**

Patients were highly satisfied with MMT services in Vietnam. However, treatment for drug users should go beyond methadone maintenance to address complicated health demands of drug users. Integrating MMT with comprehensive HIV and general health services together with improving the capacity of health workers and efficiency of services organisation to provide interconnected health care for drug users are critical for improving the outcomes of the MMT program.

## Introduction

In Vietnam, HIV concentrated epidemic is mostly attributable to people who inject drug (PWID). It is estimated that there were 180,000 drug users and over 70% of them had a history of intravenous use[1–4]. A recent study shows that the prevalences of HIV and HCV among drug users were 35.1 and 88.8%, respectively[5]. Heroin is the most widely consumed (90%) drug in the country[4, 6, 7]. Methadone maintenance treatment (MMT) is considered a standard medication for drug abuse treatment[8]. Since its first introduction in 2008, to date, approximately 31.200 illicit drug users (DUs) has received MMT in 170 nationwide clinics [4, 6, 7, 9, 10]. With the strong political will, Vietnam government has a plan to scale-up the coverage of MMT program to 80.000 DUs in the following years[10]. However, this plan has to face a challenge from a rapid cut of financial aids from international donors[10]. To address this issue, strategies to reduce operational resources and optimize the efficiency are necessary to ensure the sustainability of MMT program. This requirement raises the need to understand the performance and quality of diverse MMT services in Vietnam.

Model to deliver MMT services may vary across settings such as stand-alone or integrating with other health care services[1]. The later model includes integrations of MMT with Provincial AIDS centers, Regional Polyclinic, and District Health Centers[1, 3]. To assess the performance of health services, self-reported information about patient experience and satisfaction play an indispensable role along with traditional health outcomes [11]. In particular, the patient satisfaction helps program managers to recognize the responsiveness of service delivery by measuring whether the patients’ needs are tackled, identifying the gaps and reflecting the quality of environment or health staffs in clinics [12, 13]. Besides, in opioid dependence treatment, patient satisfaction may use to predict the retention, adherence, treatment outcome and risk of drug relapse [13–15]. Levels of satisfaction as well as their determinants varied across socio-economic factors, health status, characteristics of MMT clinics as well as dose of MMT [16–18]. Accordingly, MMT providers should learn to understand the characteristics of patient satisfaction in each setting, which is important to improve service delivery.

Currently, Vietnam has implemented MMT clinics in various models, including not only standalone- but also integrative- services. For example, MMT service may be co-located or combined with other HIV-related services such as antiretroviral treatment (ART) or HIV counselling and testing services (VCT). Likewise, it can be integrated with general health care service. However, in Vietnam, none of literature has mentioned the satisfaction of MMT patients for their attended clinics. Thus, the purpose of this paper was to examine the difference of satisfaction among MMT patients in various service delivery models and explore related factors.

## Methods

### Ethics approval

The protocol of this study was reviewed and approved by the Vietnam Authority of HIV/AIDS Control's Scientific Research Committee. Written informed consent was obtained from all participants. Patients could withdraw at any time without the influence on their current treatment.

### Study design and sampling technique

From January to August 2013, a cross-sectional survey was conducted in two Vietnamese HIV epicenters namely Hanoi and Nam Dinh. Five clinics were purposively selected in the study based on following criteria: 1) delivering MMT services; 2) representing both urban and rural areas, and 3) covering various levels of health system such as provincial- and district- levels. The detailed information of clinics is described in Table 1. It is important to notice that this study sample has a limited representativeness for the MMT patient population. We selected the two provinces in consultation with program managers at the Vietnam Authority of HIV/AIDS for a purposive comparison of an experienced setting—Hanoi and a new setting—Nam Dinh Province. Also the selection of MMT sites was primarily for the comparison of various service develivery models in different level of health administration.

**Table 1:** Study settings and sample size.

Level	Settings	Site Name	Type of services	Sample size
Province	Nam Dinh City	Provincial AIDS Centre	MMT+ VCT	270
District (rural)	Xuan Truong District	District Health Centre	MMT+ VCT + ART + GH	151
District (urban)	Tu Liem District	District Health Centre	MMT+ VCT + ART + GH	201
District (urban)	Long Bien District	District Health Centre	MMT+ VCT + ART + GH	184
District (urban)	Ha Dong District	Regional Polyclinic	MMT+ GH	210

VCT: Voluntary HIV Counseling and Testing; ART: Antiretroviral Treatment; GH: General Health; MMT: Methadone Maintenance Treatment.

We invited all patients who were present at seclected MMT clinics to participate in the study. The eligibility criteria: 1) presenting at clinics during study period; 2) being 18 years old or above; 3) having capacity to answer the questionnaire and 4) providing informed consent to participate.

Initially, patients were invited to a designated counseling room to ensure their privacy. Then, interviewer introduced the purposes of this study and the benefits to improving MMT program that in turn support the patients as they accepted to participate. Finally, we gave written informed consents to patients for signature. Data collection procedure was conducted within 15 to 30 minutes by well-trained researchers. There was none of MMT health staffs involving in this procedure. A total of 1,016 patients were recruited in the study.

### Measures and instruments

A structured questionnaire was developed for data collection. The information of concern included socio-economic factors (age, gender, education, marital, religion and employment status), health status (including HIV status) and type of MMT models. Those variables were selected based on previous studies [16–18].

EQ-5D-5L instrument was used to measure health status. Its Vietnamese version was validated elsewhere [19]. This instrument includes five dimensions (mobility, self-care, usual activities, pain/discomfort, and anxiety/depression) with five response levels, from “No problem” to “Extremely problem” [19, 20]. We categorized people who reported “Slightly” to “Extremely” into “Having problem” group, while others classified “No problem”.

To measure the patient satisfaction, we modified a previously developed instrument for Vietnamese settings, namely **Satisfaction with HIV/AIDS Treatment Interview Scale (SATIS)**. This generic instrument was used to measure the satisfaction of patients for HIV/AIDS-related services. The procedure to develop this scale was described elsewhere [21]. Generally, SATIS includes 10 items with the range of options for response being from 0 to 10, where 0 indicated complete dissatisfaction and 10 indicated complete satisfaction. The scores of specific domains were computed by averaging the score of correspond items. The higher score means the higher level of satisfaction. Additionally, the instrument comprises two global ratings of overall satisfaction with health services and treatment outcomes. This instrument was used to measure the patient satisfaction for HIV-related service delivery models in Vietnam [21]. In this study, SATIS was speficified for MMT clinics and other quality features was asked for the MMT that patients were attending while the measure items’ content, response options and scoring remained unchanged.

### Statistical analysis

In this study, we employed *exploratory factor analysis* (EFA) to explore the construct validity of the SATIS measurement. Principle component analysis was used to extract those factors. An eigenvalue of 0.35, where its curve flattened out, was selected as a threshold. The threshold was defined by the scree test. We used Orthogonal Varimax rotation with Kaisers’ normalization to re-organize items in the scale, which aimed to increase the interpretability of these factors. A value of 0.55 was utilized to be a cut-off point for factor loadings. We also performed a cross-loading in one item and then assigned it to the appropriate domain based on both the nature of the question and the overarching dimension.

Cronbach’s alpha was used to assess the internal consistency reliability of measurement. Chi-squared, t-test and ANOVA were used to explore the differences of satisfaction among characteristics. Multivariate linear and logistic regressions were employed to identify the associated factors with reclassified domains and general satisfaction. In this study, we applied a stepwise forward model strategy which using log-likelihood ratio test at a p-value of 0.1 to select variables for the reduced models [22]. A p-value < 0.05 was set as the level of statistical significance.

## Results

Table 2 shows the socio-economic status and health status of the sample. Overall, the mean age of respondents was 35.8 (SD = 7.5). The majority of patients were male (98.7%), attaining secondary school or above (86.6%) and living with spouse (67.4%). Most of the patients were cult of ancestors (88.2%) and self-employed (53.4%). Regarding to health status, the proportion of respondents having HIV-positive status was 8.1%. When only 7.3%, 3.9% and 5.9% had problems in morbidity, self-care and usual activity, respectively, about one of five respondents had pain/discomfort and anxiety/depression problems (17.7% and 20.7%, correspondingly).

**Table 2:** Socio-economic characteristics and health status of respondents.

			MMT+VCT+ART+DGH								p-value
	MMT+VCT		Rural		Urban		MMT+RPC		All		
	Mean	SD	Mean	SD	Mean	SD	Mean	SD	Mean	SD	
**Age**	36.8	7.3	36.8	8.0	36.4	7.9	37.0	7.5	35.8	7.5	0.83
	**N**	**%**	**N**	**%**	**N**	**%**	**N**	**%**	**N**	**%**	
**Sex (Male)**	266	98.5	151	100.0	206	98.1	380	98.7	1003	98.7	0.44
**Educational attainment**										
Illiterate	4	1.5	1	0.7	4	1.9	8	2.1	17	1.7	<0.01
Elementary	21	7.8	29	19.2	27	12.9	42	10.9	119	11.7	
Secondary	103	38.2	87	57.6	86	41.0	150	39.0	426	41.9	
High	121	44.8	28	18.5	81	38.6	157	40.8	387	38.1	
Vocational	12	4.4	5	3.3	7	3.3	8	2.1	32	3.2	
University	9	3.3	1	0.7	5	2.4	20	5.2	35	3.4	
**Marital status**											
Single	101	37.4	29	19.2	47	22.4	74	19.2	251	24.7	<0.01
Live with spouse	148	54.8	116	76.8	147	70.0	274	71.2	685	67.4	
Live with partner	0	0.0	0	0.0	1	0.5	2	0.5	3	0.3	
Divorced	19	7.0	6	4.0	15	7.1	32	8.3	72	7.1	
Widow	2	0.7	0	0.0	0	0.0	3	0.8	5	0.5	
**Religion**											
Cult of ancestors	247	91.5	96	63.6	198	94.3	355	92.2	896	88.2	<0.01
Buddhism	13	4.8	16	10.6	10	4.8	20	5.2	59	5.8	
Catholic	10	3.7	39	25.8	2	1.0	5	1.3	56	5.5	
Protestant	0	0.0	0	0.0	0	0.0	5	1.3	5	0.5	
**Employment**											
Unemployed	76	28.2	25	16.6	53	25.2	105	27.3	259	25.5	<0.01
Self-employed	159	58.9	67	44.4	112	53.3	204	53.0	542	53.4	
White collars	5	1.9	1	0.7	5	2.4	11	2.9	22	2.2	
Workers, Farmers	10	3.7	54	35.8	18	8.6	18	4.7	100	9.8	
Students	0	0.0	0	0.0	0	0.0	2	0.5	2	0.2	
Other jobs	20	7.4	4	2.7	22	10.5	45	11.7	91	9.0	
**Health status**											
HIV-positive status	22	8.2	7	4.6	9	4.3	44	11.4	82	8.1	<0.01
Having morbidity problem	19	7.0	20	13.3	15	7.1	20	5.2	74	7.3	<0.05
Having self-care problem	10	3.7	9	6.0	9	4.3	12	3.1	40	3.9	0.49
Having usual activity problem	18	6.7	17	11.3	9	4.3	16	4.2	60	5.9	<0.05
Having pain/discomfort problem	37	13.7	57	37.8	34	16.2	52	13.5	180	17.7	<0.01
Having anxiety/depression problem	56	20.7	65	43.1	38	18.1	51	13.3	210	20.7	<0.01

The construct validity of SATIS was displayed in Table 3. Three dimensions were reclassified from factor analysis namely “Services quality and convenience”, “Health workers’ capacity and responsiveness” and “Inter-professional care”. Those dimensions accounted for 84.3% of the variance, of which the highest share of the variance belonged to the first dimension with 33.2%. Cronbach’s alpha was excellent across domains with the range from 0.90 to 0.94.

**Table 3:** Factor loadings of SATIS measure in MMT patients.

		Factor loadings of SATIS domains		
Items	% completely satisfied	Services quality and convenience	Capacity of health workers & responsiveness	Inter-professional care
Quality of MMT services delivery	49.0	0.68		
Access to information and guidance on hospital services and procedures	51.4	0.76		
Consultation, explanation, and guidance of health care workers	53.1	0.79		
Convenience in check-up booking, waiting time, and administrative procedure	51.1	0.66		
Convenience in using related medical services within the facility, e.g. lab tests, referrals, or related examinations by different specialists	53.0			0.74
Inter-professional and inter-departmental collaborations	53.9			0.78
Competence of health care workers	54.5		0.70	
Responsiveness of health care workers to patients' questions and requests	56.1		0.75	
Availability of patients' needed health care services	53.5		0.65	
Medical confidentiality and respect of patients’ privacy	60.8		0.74	
**% floor**		49.0%	0.5%	79.0%
**% ceiling**		43.1%	47.9%	50.0%
**Reliability**				
Cronbach’s alpha		0.94	0.9342	0.9092
**SATIS domains scores**				
Mean		9.12	9.20	9.12
SD		1.19	1.19	1.37
**General satisfaction**				
% Completely satisfied with general health services	36.48			
% Completely satisfied with health outcomes	34.89			

Table 3 also showed that the proportion of patients completely satisfying was the highest in “Confidentiality” (60.8%) and “Responsiveness” (56.1%). Conversely, the proportion was the lowest in “Quality” (49.0%) and “Convenience” (51.1%). Additionally, the average score of each domain was high, with the highest in “Capacity of health workers & responsiveness” (9.20±1.19).

The average scores of each item and domain regarding to MMT delivery models were illustrated in Table 4. Overall, the patient satisfaction for “MMT+VCT” models or “MMT+VCT+ART+ General health care” in rural areas was significantly lower than those two other models (p<0.05) in specific items and three domains as well.

**Table 4:** Satisfaction with MMT by different service models.

			MMT+VCT+ART+DGH						
	MMT+VCT		Rural		Urban		MMT+RPC		p-value
SATIS measure items and domains	Mean	SD	Mean	SD	Mean	SD	Mean	SD	
Quality of MMT services delivery	8.88	1.37	8.87	1.48	9.18	1.31	9.29	0.93	<0.01
Access to information and guidance on hospital services and procedures	8.88	1.44	8.99	1.53	9.20	1.34	9.37	0.86	<0.01
Consultation, explanation, and guidance of health care workers	8.91	1.38	9.10	1.56	9.27	1.22	9.40	0.85	<0.01
Convenience in check-up booking, waiting time, and administrative procedure	8.82	1.43	9.03	1.46	9.23	1.27	9.20	1.15	<0.01
Convenience in using related medical services within the facility, e.g. lab tests, referrals, or related examinations by different specialists	8.91	1.44	9.07	1.51	9.19	1.50	9.28	1.19	0.02
Inter-professional and inter-departmental collaborations	8.82	1.72	9.05	1.56	9.21	1.42	9.38	0.93	<0.01
Competence of health care workers	8.89	1.44	9.12	1.54	9.25	1.38	9.31	1.12	<0.01
Responsiveness of health care workers to patients' questions and requests	8.91	1.45	9.29	1.21	9.20	1.49	9.44	0.84	<0.01
Availability of patients' needed health care services	8.90	1.46	9.16	1.35	9.22	1.42	9.35	0.94	<0.01
Medical confidentiality and respect of patients’ privacy	9.13	1.33	9.23	1.55	9.38	1.25	9.41	0.97	0.04
**SATIS domains**									
Services quality and convenience	8.88	1.26	8.99	1.35	9.22	1.21	9.32	0.87	<0.01
Capacity health workers & responsiveness	8.86	1.51	9.06	1.49	9.20	1.38	9.33	0.98	<0.01
Inter-professional care	8.96	1.28	9.20	1.23	9.26	1.24	9.38	0.87	<0.01
**Overall SATIS Score**	8.92	1.24	9.08	1.28	9.23	1.21	9.34	0.83	<0.01

DGH: District General Health Center; RPC: Regional PolyClinics.

Table 5 presents the relations between SATIS domains and patients’ characteristics in reduced multivariate models. The finding indicates that compared to MMT+VCT models, three other MMT delivery models were positively associated with the satisfaction across SATIS domains. Patients in those clinics were more likely to be completely satisfied with quality of services and treatment outcomes. In addition, some specific characteristics were related with each SATIS domain. For example, people at age group 40–45, attaining high school education and having self-care problems were found a small to large decrements in “Services quality and convenience” and “Inter-professional care”. Moreover, we also found a small decrement in the last domain among patients having anxiety/depression problem.

**Table 5:** Factors associated with patients’ satisfaction with Methadone Maintenance Treatment.

	SATIS domain scores						Complete satisfaction with			
	Services quality and convenience		Capacity health workers & responsiveness		Inter-professional care		MMT services		Health Outcome	
	Coef	SE	Coef	SE	Coef	SE	OR	SE	OR	SE
**MMT model (MMT+VCT—ref)**										
Rural MMT-ART-VCT-DGH	0.096	0.152	0.13	0.18	0.321*	0.151	2.90**	0.87	2.61**	0.80
Urban MMT-ART-VCT-DGH	0.321**	0.104	0.17	0.12	0.247*	0.103	2.47**	0.53	2.11**	0.46
MMT + Regional poly clinic	0.432**	0.120	0.35**	0.14	0.423**	0.117	2.66**	0.63	2.02**	0.50
**HIV status**										
Not reported vs. Negative							3.34**	1.40	2.74**	1.13
**Age group (18-<25—ref)**										
25-<30							0.63	0.15		
35- <40									1.43	0.27
40- <45	-0.282*	0.122	-0.270	0.139	-0.287*	0.119				
**Education (Illiterate—ref)**										
Elementary									0.19*	0.14
Secondary									0.09**	0.06
High	-0.219*	0.089	-0.198	0.101	-0.222*	0.088	0.64*	0.11	0.07**	0.05
Vocational									0.09**	0.07
University					-0.413	0.233			0.11**	0.09
**Marital status (Single—ref)**										
Divorced							1.76	0.53		
**Employment (Unemployed—ref)**										
Other jobs							0.54	0.17	0.33**	0.12
**Reported health problems (vs. No)**										
Self-care	-0.655*	0.214	-0.482	0.249	-0.547*	0.214				
Anxiety or Depression			-0.211	0.128	-0.253*	0.109	0.41**	0.10	0.32**	0.08
Constant	8.994**	0.088	9.108**	0.104	9.149**	0.090	0.38**	0.07	3.24	2.35

* significant at 5% level;

** significant at 1% level.

In term of general quality of MMT services, being high school and having anxiety/depression problems were negatively related with completely satisfy, when people with unknown HIV status were more likely to completely satisfy. These patients were also more likely to complete satisfy with treatment outcome. Meanwhile, higher education, having other jobs and having anxiety/depression problems were inversely related with being completely satisfied.

## Discussion

This article presents the findings of a large patient satisfaction survey for MMT clinics in Vietnam. The result shows a high level of patient satisfaction across three domains namely “Services quality and convenience”, “Health workers’ capacity and responsiveness” and “Inter-professional care”, suggesting the acceptance for the quality of services. More specifically, “Confidentially” and “Responsiveness” were observed the highest proportion of people being completely satisfied in comparison to other aspects. The determinants of satisfaction included MMT delivery models, socio-economic factors (age, education, marital and employment status), health-related quality of life and HIV status.

This study highlights a tremedous effort of the Government of Vietnam in response to the epidemics of HIV and substance abuse. Not only that MMT services have been rapidly scaled up, but also the quality of these services have been improved[23, 24]. As demonstrated, “Quality” (measuring the overall quality of services) and “Convenience” (mentioning waiting time and administrative procedure) had the lowest proportion of patients being completely satisfied compared to other aspects, which were consistent with previous surveys [25, 26]. Those findings were also similar to the results for other diseases-related services such as HIV [21, 27, 28], type 2 diabetes/hypertension [29] and hospital admission [30]. It suggested that the administrative procedure and waiting time remained as barriers for accessing MMT services. Likewise, 51.4% and 53.1% patients felt completely satisfaction with the accessibility of information and the consultation of health workers, respectively, indicating that drug users might receive deficient and less responsive information for their treatment from providers. These figures were significantly lower than in Malaysia where 85% patients completed satisfied with overall treament[18].

When investigating the determinants of patient satisfaction, the result showed that higher levels of education were associated with being dissatisfied across SATIS domains. This phenomenon was common among various populations in diverse healthcare settings [16, 31]. It is because people with high education had higher expectations and requirement for the services; thus they did not easily satisfy with the quality of services. The similar tendency was observed in people having jobs or being higher age, suggesting that health staffs should provide tailored treatment for specific subjects to maximize the satisfaction of those patients. This is also in line with findings from a survey in Spain by Trujols et al. where social functiong was possitively associated with higher satisfaction with MMT[16]. In Malaysia, waiting area and staff shortages are major barriers, however, in Vietnam these are not factors associated with patients’ satisfaction given that all MMT facilities are organized following the national guideline with designated rooms and sufficient staffs.[18]

Of note, we found that the level of satisfaction of MMT patients in MMT clinics without other healthcare services was significantly lower than that in other clinics. Moreover, people with self-care and anxiety/depression tended to dissatisfy in all SATIS domains as well as general quality of services and treatment outcome. The results reflect that drug users preferred for the comprehensive care clinics, include services for physical, psychological and HIV-related care, rather than only MMT treatment services (or with VCT services). Drug users might exposure to high risk HIV-related behaviours such as alcohol drinking and unsafe sexual activities [32, 33], requiring the frequent use of counselling and care [34]. In the context of HIV epidemic driven by PWIDs, combining health services care into a single site is important to improve the accessibility of patients [35–37]. On the other hand, for HIV-positive drug users, MMT service integrates with ART could facilitate the HIV treatment adherence and outcome [2].

This study contributes to the understanding of measurement properties of the EQ-5D-5L in drug-using populations. Compared to previous studies, the percentage of having psychological health problems in this sample was lower than in HIV population and higher than general population[19, 34]. It is important to notice that EQ-5D-5L is a generic health measure that may result in a high ceiling effect and should be considered in repeated measurements[19, 34, 38].

To date, this is the initial study to provide the evidence about patient satisfaction among MMT patients in diverse type of MMT delivery models. The results would partly contribute to the expansion of MMT program in Vietnam. Findings of this study suggested several implications. First, integrating MMT with other health services such as HIV-related services and especially general health service is critical that should be implemented instead of stand-alone model. This model is not only for reducing operational cost but promptly meeting the needs of patients for comprehensive care [39–42]. Second, the administrative procedures should be simplified and the flow of services should be organized in appropriate approaches to reduce the inconvenience of patients. Besides, the linkages and referrals among related services for drug users should also be improved along with providing sufficient information and guidance for patients. Third, capacity building for health workers should be under consideration to improve the ability to provide responsive consultation and guidance for MMT patients during treatment. Finally, implementing MMT program should be incorporated with collecting data of patient satisfaction. Identifying promptly the expectations and the response of patients could help to identify the gaps of health services, enhance the quality of care and eventually promote treatment outcomes of patients [25].

The strengths of this study comprise the utilization of validated instruments to measure the patient satisfaction with drug use treatment as well as the quality of services. In addition, the survey involved the large number of MMT patients in various settings. However, there are several limitations should be under consideration. First, the causal relations were not drawn with the application of cross-sectional design. Furthermore, convenient sampling technique might limit the representative for the population of MMT patients and the capacity to generalize the findings to the whole population. We would also acknowledge the limitation that this analysis did not involve factors such as HCV status, severity of addiction; concomitant substance use, and methadone dose, given the limitation of a service users survey. In Baltimore, Kelly et al. found that satisfaction with counselors and MMT program predict the decrease in addiction severity and drop-outs[15]. Although some studies have shown that HCV status and serverity of addiction and MMT dose are positively corrected with health-related quality of life—a measure of health outcome that we used, future studies should incorporate specific drug-related measures[16, 43–46].

In conclusion, the study indicated the high level of satisfaction among MMT patients at various MMT delivery models. However, treatment for drug users should go beyond methadone maintenance to address complicated health demands of drug users. Integrating MMT with comprehensive HIV and general health services, a long with improving the capacity of health workers and efficiency of services organisation to provide interconnected health care for drug users are critical for improving the outcomes of the MMT program.

## References

[1] Tran BX, Nguyen LH, Phan HT, Nguyen LK, Latkin CA. Preference of methadone maintenance patients for the integrative and decentralized service delivery models in Vietnam. Harm reduction journal. 2015;12(1):29 Epub 2015/09/18. 10.1186/s12954-015-0063-0 26377824 PMC4574353

[2] Tran BX, Ohinmaa A, Duong AT, Nguyen LT, Vu PX, Mills S, et al Cost-effectiveness of integrating methadone maintenance and antiretroviral treatment for HIV-positive drug users in Vietnam's injection-driven HIV epidemics. Drug and alcohol dependence. 2012;125(3):260–6. Epub 2012/03/23. 10.1016/j.drugalcdep.2012.02.021 . 22436971

[3] Tran BX, Ohinmaa A, Duong AT, Nguyen LT, Vu PX, Mills S, et al The cost-effectiveness and budget impact of Vietnam's methadone maintenance treatment programme in HIV prevention and treatment among injection drug users. Global public health. 2012;7(10):1080–94. Epub 2012/10/31. 10.1080/17441692.2012.736259 . 23106230

[4] Tran BX, Ohinmaa A, Duong AT, Do NT, Nguyen LT, Nguyen QC, et al Changes in drug use are associated with health-related quality of life improvements among methadone maintenance patients with HIV/AIDS. Qual Life Res. 2012;21(4):613–23. Epub 2011/07/07. 10.1007/s11136-011-9963-y . 21732198

[5] Zhang L, Celentano DD, Le Minh N, Latkin CA, Mehta SH, Frangakis C, et al Prevalence and correlates of HCV monoinfection and HIV and HCV coinfection among persons who inject drugs in Vietnam. European journal of gastroenterology & hepatology. 2015;27(5):550–6. Epub 2015/03/15. 10.1097/MEG.0000000000000321 25769097 PMC4380662

[6] Tran BX. Willingness to pay for methadone maintenance treatment in Vietnamese epicentres of injection-drug-driven HIV infection. Bulletin of the World Health Organization. 2013;91(7):475–82. Epub 2013/07/05. 10.2471/BLT.12.115147 23825874 PMC3699795

[7] Tran BX, Ohinmaa A, Mills S, Duong AT, Nguyen LT, Jacobs P, et al Multilevel predictors of concurrent opioid use during methadone maintenance treatment among drug users with HIV/AIDS. PloS one. 2012;7(12):e51569 Epub 2012/12/20. 10.1371/journal.pone.0051569 23251580 PMC3520938

[8] Decision 1001/QD-TTg on National Strategy on Drug Control, (2011).

[9] Tran BX, Ohinmaa A, Duong AT, Do NT, Nguyen LT, Mills S, et al Cost-effectiveness of methadone maintenance treatment for HIV-positive drug users in Vietnam. AIDS Care. 2012;24(3):283–90. Epub 2011/09/23. 10.1080/09540121.2011.608420 . 21936718

[10] Control VAoHA. The annual review of HIV/AIDS control and prevention in the first six months 2015 and action plan in the last six months in 2015. Hanoi: Ministry of Health, 2014.

[11] Smith PC, Mossialos E, Papanicolas I. Performance measurement for health system improvement: experiences, challenges and prospects. Copenhagen, Denmark: World Health Organization, 2008.

[12] Simpson DD, Joe GW, Rowan-Szal GA, Greener JM. Drug abuse treatment process components that improve retention. Journal of substance abuse treatment. 1997;14(6):565–72. Epub 1998/01/23. . 9437628 10.1016/s0740-5472(97)00181-5

[13] Zhang Z, Gerstein DR, Friedmann PD. Patient Satisfaction and Sustained Outcomes of Drug Abuse Treatment. Journal of health psychology. 2008;13(3):388–400. 10.1177/1359105307088142 ; PubMed Central PMCID: PMCPmc2796692. 18420772 PMC2796692

[14] McLellan AT, Woody GE, Metzger D, McKay J, Durrell J, Alterman AI, et al Evaluating the effectiveness of addiction treatments: reasonable expectations, appropriate comparisons. The Milbank quarterly. 1996;74(1):51–85. Epub 1996/01/01. . 8596524

[15] Kelly SM, O'Grady KE, Brown BS, Mitchell SG, Schwartz RP. The role of patient satisfaction in methadone treatment. Am J Drug Alcohol Abuse. 2010;36(3):150–4. Epub 2010/05/15. 10.3109/00952991003736371 20465372 PMC2938880

[16] Trujols J, Garijo I, Sinol N, del Pozo J, Portella MJ, Perez de los Cobos J. Patient satisfaction with methadone maintenance treatment: the relevance of participation in treatment and social functioning. Drug and alcohol dependence. 2012;123(1–3):41–7. 10.1016/j.drugalcdep.2011.10.014 . 22071121

[17] Perez de los Cobos J, Fidel G, Escuder G, Haro G, Sanchez N, Pascual C, et al A satisfaction survey of opioid-dependent clients at methadone treatment centres in Spain. Drug Alcohol Depend. 2004;73(3):307–13. Epub 2004/03/24. 10.1016/j.drugalcdep.2003.11.001 . 15036553

[18] Aziz Z, Chong NJ. A satisfaction survey of opioid-dependent patients with methadone maintenance treatment. Journal of substance abuse treatment. 2015;53:47–51. Epub 2015/01/27. 10.1016/j.jsat.2014.12.008 . 25616750

[19] Tran BX, Ohinmaa A, Nguyen LT. Quality of life profile and psychometric properties of the EQ-5D-5L in HIV/AIDS patients. Health Qual Life Outcomes. 2012;10:132 Epub 2012/11/03. 10.1186/1477-7525-10-132 23116130 PMC3541089

[20] EQOL G. EQ-5D-5L User Guide: Basic information on how to use the EQ-5D-5L instrument. Rotterdam, The Netherlands 2011.

[21] Tran BX, Nguyen NP. Patient satisfaction with HIV/AIDS care and treatment in the decentralization of services delivery in Vietnam. PloS one. 2012;7(10):e46680 10.1371/journal.pone.0046680 23071611 PMC3465274

[22] Hosmer DW Jr., L S, Sturdivant RX. Applied Logistic Regression. 3rd ed: Wiley; 2013.

[23] Nguyen TT, Nguyen LT, Pham MD, Vu HH, Mulvey KP. Methadone maintenance therapy in Vietnam: an overview and scaling-up plan. Advances in preventive medicine. 2012;2012:732484 Epub 2012/12/12. 10.1155/2012/732484 23227351 PMC3512212

[24] Jardine M, Thi Nguyen VA, Khuat TH. Case study: Methadone maintenance treatment in Hanoi, Vietnam. Harm reduction journal. 2012;9:26 Epub 2012/07/10. 10.1186/1477-7517-9-26 22769568 PMC3423062

[25] Hogan B, Hershey L, Ritchey S. A case study using a patient satisfaction survey to improve the delivery and effectiveness of drug addiction treatment services: marketing implications and organizational impact. Health marketing quarterly. 2007;24(1–2):93–106. Epub 2008/12/02. 10.1080/07359680802125923 . 19042522

[26] Madden A, Lea T, Bath N, Winstock AR. Satisfaction guaranteed? What clients on methadone and buprenorphine think about their treatment. Drug Alcohol Rev. 2008;27(6):671–8. Epub 2009/04/21. 10.1080/09595230801935706 . 19378450

[27] Chow MY, Li M, Quine S. Client satisfaction and unmet needs assessment: evaluation of an HIV ambulatory health care facility in Sydney, Australia. Asia-Pacific journal of public health / Asia-Pacific Academic Consortium for Public Health. 2012;24(2):406–14. 10.1177/1010539510384843 . 21118857

[28] Olowookere SA, Fatiregun AA, Ladipo MM, Akenova YA. Reducing waiting time at a Nigerian HIV treatment clinic: opinions from and the satisfaction of people living with HIV/AIDS. Journal of the International Association of Physicians in AIDS Care (Chicago, Ill: 2002). 2012;11(3):188–91. Epub 2011/04/28. 10.1177/1545109711402214 . 21521808

[29] Doubova SV, Perez-Cuevas R, Zepeda-Arias M, Flores-Hernandez S. Satisfaction of patients suffering from type 2 diabetes and/or hypertension with care offered in family medicine clinics in Mexico. Salud publica de Mexico. 2009;51(3):231–9. Epub 2009/12/08. . 19967309 10.1590/s0036-36342009000300014

[30] Mitike G, Mekonnen A, Osman M. Satisfaction on outpatient services in hospitals of the Amhara Region. Ethiopian medical journal. 2002;40(4):387–96. Epub 2003/02/25. . 12596658

[31] Crow R, Gage H, Hampson S, Hart J, Kimber A, Storey L, et al The measurement of satisfaction with healthcare: implications for practice from a systematic review of the literature. Health technology assessment (Winchester, England). 2002;6(32):1–244. Epub 2003/08/20. . 12925269 10.3310/hta6320

[32] Stein MD, Charuvastra A, Anderson B, Sobota M, Friedmanna PD. Alcohol and HIV risk taking among intravenous drug users. Addictive behaviors. 2002;27(5):727–36. Epub 2002/08/31. . 12201380 10.1016/s0306-4603(01)00205-2

[33] Donoghoe MC. Sex, HIV and the injecting drug user. British journal of addiction. 1992;87(3):405–16. Epub 1992/03/01. . 1559039 10.1111/j.1360-0443.1992.tb01941.x

[34] Tran BX, Ohinmaa A, Nguyen LT, Nguyen TA, Nguyen TH. Determinants of health-related quality of life in adults living with HIV in Vietnam. AIDS care. 2011;23(10):1236–45. Epub 2011/06/30. 10.1080/09540121.2011.555749 . 21711211

[35] Organization WH. Treatment of injecting drug users with HIV/AIDS: promoting access and optimizing service delivery. Geneva, Switzerland: 2006.

[36] Samet JH, Stein MD, O'Connor PG. Models of Medical Care for HIV-infected Drug Users. Substance Abuse. 1995;16(3):131–9. 10.1080/08897079509444716

[37] Herman M, Gourevitch MN. Integrating primary care and methadone maintenance treatment: implementation issues. Journal of addictive diseases. 1997;16(1):91–102. 10.1300/J069v16n01_06 . 9046446

[38] Tran B, Nguyen L, Ohinmaa A, Maher R, Nong V, Latkin CA. Longitudinal and cross sectional assessments of health utility in adults with HIV/AIDS: a systematic review and meta-analysis. BMC Health Serv Res. 2015;15(1):7 Epub 2015/01/23. 10.1186/s12913-014-0640-z 25609449 PMC4307193

[39] van Beek I. Case study: accessible primary health care—a foundation to improve health outcomes for people who inject drugs. The International journal on drug policy. 2007;18(4):329–32. 10.1016/j.drugpo.2006.11.005 . 17689383

[40] O'Connor PG, Molde S, Henry S, Shockcor WT, Schottenfeld RS. Human immunodeficiency virus infection in intravenous drug users: a model for primary care. The American journal of medicine. 1992;93(4):382–6. . 1415300 10.1016/0002-9343(92)90166-9

[41] Selwyn PA, Budner NS, Wasserman WC, Arno PS. Utilization of on-site primary care services by HIV-seropositive and seronegative drug users in a methadone maintenance program. Public Health Reports. 1993;108(4):492–500. PMC1403415. 8393579 PMC1403415

[42] Umbricht-Schneiter A, Ginn DH, Pabst KM, Bigelow GE. Providing medical care to methadone clinic patients: referral vs on-site care. American Journal of Public Health. 1994;84(2):207–10. PMC1615007. 8296941 10.2105/ajph.84.2.207 PMC1615007

[43] Marchand K, Palis H, Peng D, Fikowski J, Harrison S, Spittal P, et al The Role of Gender in Factors Associated With Addiction Treatment Satisfaction Among Long-Term Opioid Users. Journal of addiction medicine. 2015 Epub 2015/09/04. 10.1097/ADM.0000000000000145 . 26335006 PMC4605272

[44] Marchand KI, Oviedo-Joekes E, Guh D, Brissette S, Marsh DC, Schechter MT. Client satisfaction among participants in a randomized trial comparing oral methadone and injectable diacetylmorphine for long-term opioid-dependency. BMC Health Serv Res. 2011;11:174 Epub 2011/07/28. 10.1186/1472-6963-11-174 21791093 PMC3161847

[45] Trujols J, Sinol N, de los Cobos JP. Methadone maintenance treatment: the need to distinguish between holding dose, dose adequacy, satisfaction with methadone as a medication, and satisfaction with treatment. Journal of clinical psychopharmacology. 2010;30(1):95–6; author reply 6. Epub 2010/01/16. 10.1097/JCP.0b013e3181c8b439 . 20075667

[46] Perez de Los Cobos J, Trujols J, Valderrama JC, Valero S, Puig T. Patient perspectives on methadone maintenance treatment in the Valencia Region: dose adjustment, participation in dosage regulation, and satisfaction with treatment. Drug Alcohol Depend. 2005;79(3):405–12. Epub 2005/05/05. 10.1016/j.drugalcdep.2005.03.021 . 15869846

